# Noradrenergic Activation of Hypoglossal Nucleus Modulates the Central Regulation of Genioglossus in Chronic Intermittent Hypoxic Rats

**DOI:** 10.3389/fneur.2017.00171

**Published:** 2017-05-01

**Authors:** Xinshi Nie, Ling Zhou, Aidi Wang, Hongyu Jin, Zheng Qin, Jian Pang, Wei Wang, Jian Kang

**Affiliations:** ^1^Institute of Respiratory Disease, The First Hospital of China Medical University, Shenyang, China; ^2^The 463rd Hospital of the Chinese PLA, Shenyang, China

**Keywords:** norepinephrine, genioglossus, hypoglossal nucleus, transcranial magnetic stimulation, obstructive sleep apnea, α1-adrenergic antagonist, central regulation

## Abstract

Neuromuscular compensation of the genioglossus muscle can be induced by chronic intermittent hypoxia (CIH) in obstructive sleep apnea to maintain upper airway stability. Noradrenergic activation of hypoglossal nucleus plays a critical role in the central control of the genioglossus. However, it remains unknown whether norepinephrine takes part in the central regulation of the genioglossus during CIH. Adult male Wistar rats (*n* = 32) were studied to explore the influence of noradrenergic activation of hypoglossal nucleus on the central control of the genioglossus at different stages of CIH. The rats were divided into four groups: normal control or normoxic (NO) group, CIH group, CIH + normal saline (NS) group, and CIH + prazosin (PZ, α1-adrenergic antagonist) group. PZ (0.2 mM, 60 nl) and NS (0.9%, 60 nl) were microinjected into the hypoglossal nucleus. The responses of the genioglossus corticomotor area to transcranial magnetic stimulation (TMS) were recorded on the 1st, 7th, 14th, and 21st day of CIH. The CIH group showed significantly shorter TMS latencies on days 1, 7, and 14 (3.85 ± 0.37 vs. 4.58 ± 0.42, 3.93 ± 0.17 vs. 4.49 ± 0.55, 3.79 ± 0.38 vs. 4.39 ± 0.30 ms, *P* < 0.05), and higher TMS amplitudes on day 1 (2.74 ± 0.87 vs. 1.60 ± 0.52 mV, *P* < 0.05) of CIH than the NO group. Compared to the CIH + NS group, the CIH + PZ group showed decreased TMS responses (longer latencies and lower amplitudes) only on the 14th day of CIH (3.99 ± 0.28 vs. 4.61 ± 0.48 ms, 2.51 ± 0.67 vs. 1.18 ± 0.62 mV, *P* < 0.05). These results indicated that noradrenergic activation of the hypoglossal nucleus played a role in the central compensation of genioglossus through α1-adrenoceptor on the 14th day of CIH.

## Introduction

Obstructive sleep apnea (OSA) is a common disease characterized by recurrent upper airway collapse and chronic intermittent hypoxia (CIH) during sleep, but not during wakefulness. In normal situations, upper airway patency depends on the balance between negative airway pressure and neuromuscular activities. The contraction of upper airway dilator muscles, such as the genioglossus, plays an important role in keeping the upper air way open (1). It has been reported that genioglossus atonia can induce upper airway collapse in rapid eye movement (REM) sleep (2). Compared to normal subjects, OSA patients showed increased activities of genioglossus electromyogram (EMG) (3) and corticomotor area during wakefulness (4). These findings indicated that OSA patients had neuromuscular compensation, which was responsible for the disappearance of apnea during daytime. Bradford et al. found that episodic hypoxia-evoked persistent increase in genioglossus EMG activity in neonatal rats *in vivo* (5). A recent study showed that CIH could elicit beneficial outcomes by initiating respiratory plasticity (6). Our previous studies also revealed increased genioglossus corticomotor activity of rats during CIH (7, 8). The above results suggested that CIH could induce the central compensation of genioglossus. However, it remains unknown how CIH influences the central control of genioglossus.

The study by Fenik et al. revealed that the combined withdrawal of norepinephrine (NE) and 5-hydroxytryptamine (5-HT) in hypoglossal nucleus could lead to the suppression of hypoglossal motoneuron during REM sleep (9). Neverova et al. reported that both 5-HT and NE were required for full expression of hypoglossal long-term facilitation induced by acute intermittent hypoxia (10). Our previous study on rats demonstrated that 5-HT in the raphe nucleus modulated the facilitation of genioglossus corticomotor activity induced by CIH (8). On the other hand, acute intermittent hypoxia-induced hypoglossal long-term facilitation could be blocked by intravenous injection of the α1-adrenergic antagonist, prazosin (PZ) (6, 11). Stettner et al. also found that PZ microinjection into the hypoglossal nucleus could cause a greater decrease of hypoglossal nerve activity in rats after the 35th day of hypoxia than those in air (12). Therefore, it is reasonable to hypothesize that noradrenergic activation of hypoglossal nucleus probably plays an excitatory role in the central control of the genioglossus during CIH. Transcranial magnetic stimulation (TMS) induced increased genioglossus neuromuscular activities in wakeful OSA patients (4, 13) and CIH rats (7, 8). With this technique, we aimed to further investigate whether noradrenergic activation of the hypoglossal nucleus played a role in the central regulation of genioglossus in rats during CIH, especially at different stages of CIH.

## Materials and Methods

### Animals

Adult male Wistar rats (250–280 g) were obtained from Liao Ning Chang Sheng Biotechnology Co., Ltd. They were randomly divided into four groups: normal control or normoxic (NO) group (*n* = 8), CIH group (*n* = 8), CIH with normal saline (NS) microinjection (CIH + NS) group (*n* = 8), and CIH with PZ microinjection (CIH + PZ) group (*n* = 8). All animals were housed in polypropylene cages with a capacity of 15 cm × 20 cm × 20 cm. They lived in controlled conditions (temperature, 24 ± 2°C, relative air humidity, 40%), of a 12:12 h light:dark cycle (8:00 a.m. to 8:00 p.m.). Standard chow diet and water were provided *ad libitum*.

### Surgery and Drug Administration

Five days before the experiment, rats in the PZ and NS microinjection groups were subjected to general anesthesia by inhalation of isoflurane (0.2–2%) and placed in a stereotaxic apparatus (68003, RWD Life Science, China). The heads were shaved and skins sterilized with alcohol and then incised. A guide cannula (62004, RWD Life Science, China) for microinjection was lowered through a burr hole into the ventral region of unilateral hypoglossal nucleus [defined by the following stereotaxic coordinates: 13.2–14.5 mm posterior to bregma, 0.1–0.3 mm lateral to the midline, 9.0–9.2 mm below the dorsal surface of the skull (14)] and fixed onto the skull with dental screw and cement. The rats were then allowed to recover from surgery. At the beginning of PZ and NS microinjections, the injection cannula (62204, RWD Life Science, China) was inserted into the guide cannula, and then connected to a 0.5-µl microsyringe (Eppendorf, German) by a polyethylene tube. PZ (α1-adrenoreceptor antagonist, 0.2 mmol/l, 60 nl, Sigma-Aldrich) and NS (0.9%, 60 nl) were, respectively, microinjected into the hypoglossal nucleus by a microinjector machine (model 310, Stoelting, IL, USA) on the 1st, 7th, 14th, and 21st day of CIH and NO. Each injection lasted 30 s. The injection cannula was left *in situ* for an additional 3 min before removal to minimize suctioning back of the drug solution into the guide cannula. TMS was carried out after microinjections.

### Chronic Intermittent Hypoxia

The rats subjected to CIH were placed in oxycycler (Oxycycler model A48XOV, BioSherix, NY, USA) with 188 s cycle of normoxia (21% O_2_ for 60 s) and hypoxia (10% O_2_ for 45 s), 8 h/day (from 8:00 a.m. to 4:00 p.m.) for three consecutive weeks. The rats in the NO group were subjected to alternating cycles of air in identical experimental conditions in parallel. The O_2_ concentration was continuously measured by an O_2_ analyzer and was modulated by controlling gas outlets using a computerized system.

### Transcranial Magnetic Stimulation

Transcranial magnetic stimulation was carried out on the 1st, 7th, 14th, and 21st day of the experiments in all groups. The rats were appropriately anesthetized with 10% chloral hydrate and positioned on wooden boards with heads, bodies, and limbs restrained. A concentric needle electrode (NM-131 T, NIHON, Kohden, Japan) was inserted into the genioglossus. Magstim 200 stimulator was used to deliver the stimulus (Magstim 200, UK). According to previous studies, the coil was held against the rat’s head, and the optimum stimulation site was 3.0–5.0 mm rostral to bregma and 2.0–4.0 mm lateral from the midline (7, 8). Five single stimulations were given with 30 s intervals, and the mean value was counted. The response of the genioglossus corticomotor area to TMS was evaluated by motor-evoked potentials (MEPs). MEP was recorded using a computer software package (AxoScope software 9.0, Axon Instruments, Inc., USA). MEP latency was defined as the time up to the first deflection from baseline following TMS, and its amplitude was measured from peak-to-peak TMS response. The methodology is described in detail in our previous studies (7, 8).

### Verification of the Microinjection Site

At the end of the experiment, the rats were deeply anesthetized and intracardially perfused with 200-ml cold saline and 500-ml cold 4% paraformaldehyde (4% in 0.1 mol/l phosphate-buffered saline, pH = 7.4). Evan’s blue (6 µl, Sigma) was injected into the hypoglossal nucleus through the injection cannula. The brains were removed and post-fixed in 4% paraformaldehyde overnight at 4°C and dehydrated in ethanol. They were cleared in dimethylbenzene and embedded in paraffin. They were then sectioned at 5 µm coronally with a microtome (RM2015, Leica) and stained with neural red to verify the localization of the injection site.

### Statistical Analysis

Statistical analysis was performed using the SPSS 17.0 for Windows. Values were reported as means ± SD. Unpaired *t*-test was used in two independent series of specimen data. For the analysis of genioglossus MEP latencies and amplitudes among different groups, repeated measures of analysis of variance (ANOVA) were used to compare the latency and amplitude of the experiment. Multivariate analysis of variance was used to make comparisons between groups at each time point. Statistical differences between multiple groups were compared using one-way ANOVA. The Duncan test was performed for multiple comparisons. *P* < 0.05 was considered statistically significant.

## Results

### Microinjection Site

**Figure 1:**
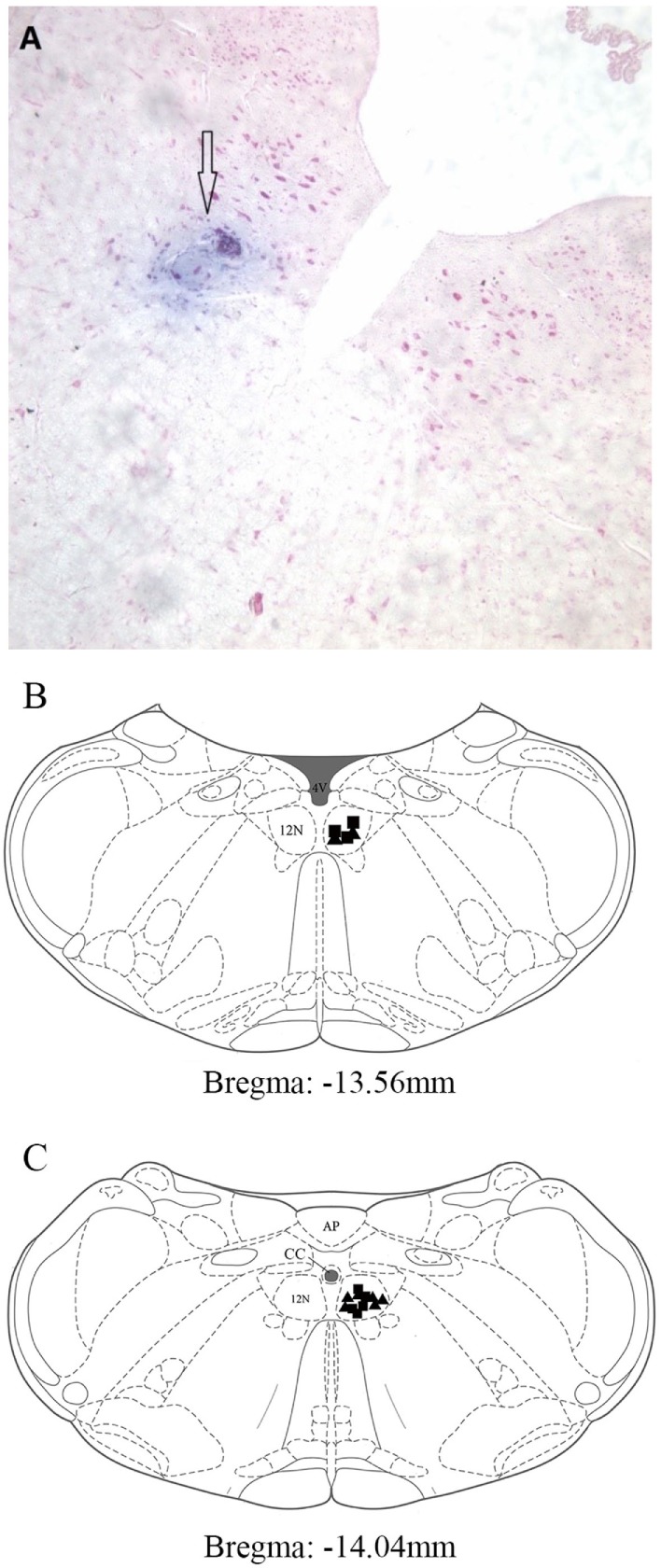
**Distribution of microinjection sites in the XII nucleus**. **(A)** A typical area stained by Evan’s blue was shown with hollow arrow. It was injected into hypoglossal nucleus, and the section was stained with Neutral Red. **(B,C)** Microinjection sites in pontomedullary superimposed into two sections from the rat brain atlas (14). The closed triangles represent the prazosin injection sites (*n* = 8). The closed squares represent the normal saline injection sites (*n* = 8). Abbreviations: AP, area postrema; CC, central canal; 4V, 4th ventricle; 12N, hypoglossal nucleus.

Figure 1A shows a typical microinjected area stained with Evan’s blue. The typical microinjection sites in the hypoglossal nucleus are shown in Figures 1B,C. All injection sites were located within the hypoglossal nucleus.

### TMS Responses of the CIH and NO Groups

**Figure 2:**
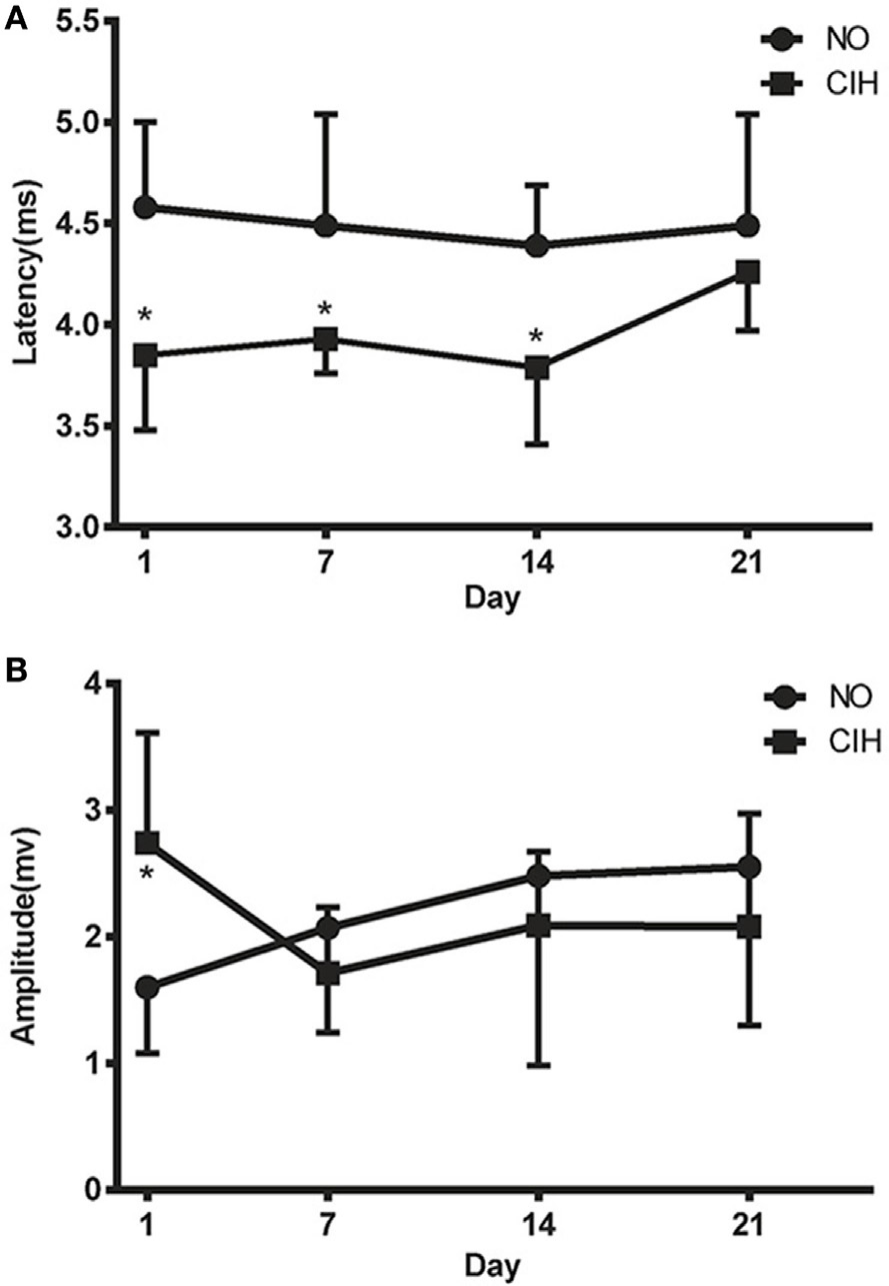
**The motor-evoked potential latencies (A) and amplitudes (B) of genioglossus corticomotor area in chronic intermittent hypoxia (CIH) and normoxic (NO) groups**. * indicates significant differences between NO and CIH groups (*P* < 0.05).

The CIH group showed significantly shorter MEP latencies than the NO group on the 1st (3.85 ± 0.37 vs. 4.58 ± 0.42 ms, *P* < 0.05), 7th (3.93 ± 0.17 vs. 4.49 ± 0.55 ms, *P* < 0.05), and 14th (3.79 ± 0.38 vs. 4.39 ± 0.30 ms, *P* < 0.05) day of exposure (Figure 2A). Significant difference in TMS amplitudes between CIH and NO rats was observed on the 1st day (2.74 ± 0.87 vs. 1.60 ± 0.52 mV, *P* < 0.05) (Figure 2B). On the 21st day, no statistically significant difference was observed in TMS responses between the NO and CIH groups. These results indicated that CIH increased genioglossus corticomotor excitability during the first 2 weeks of exposure.

### TMS Responses of the CIH + PZ and CIH + NS Groups

**Figure 3:**
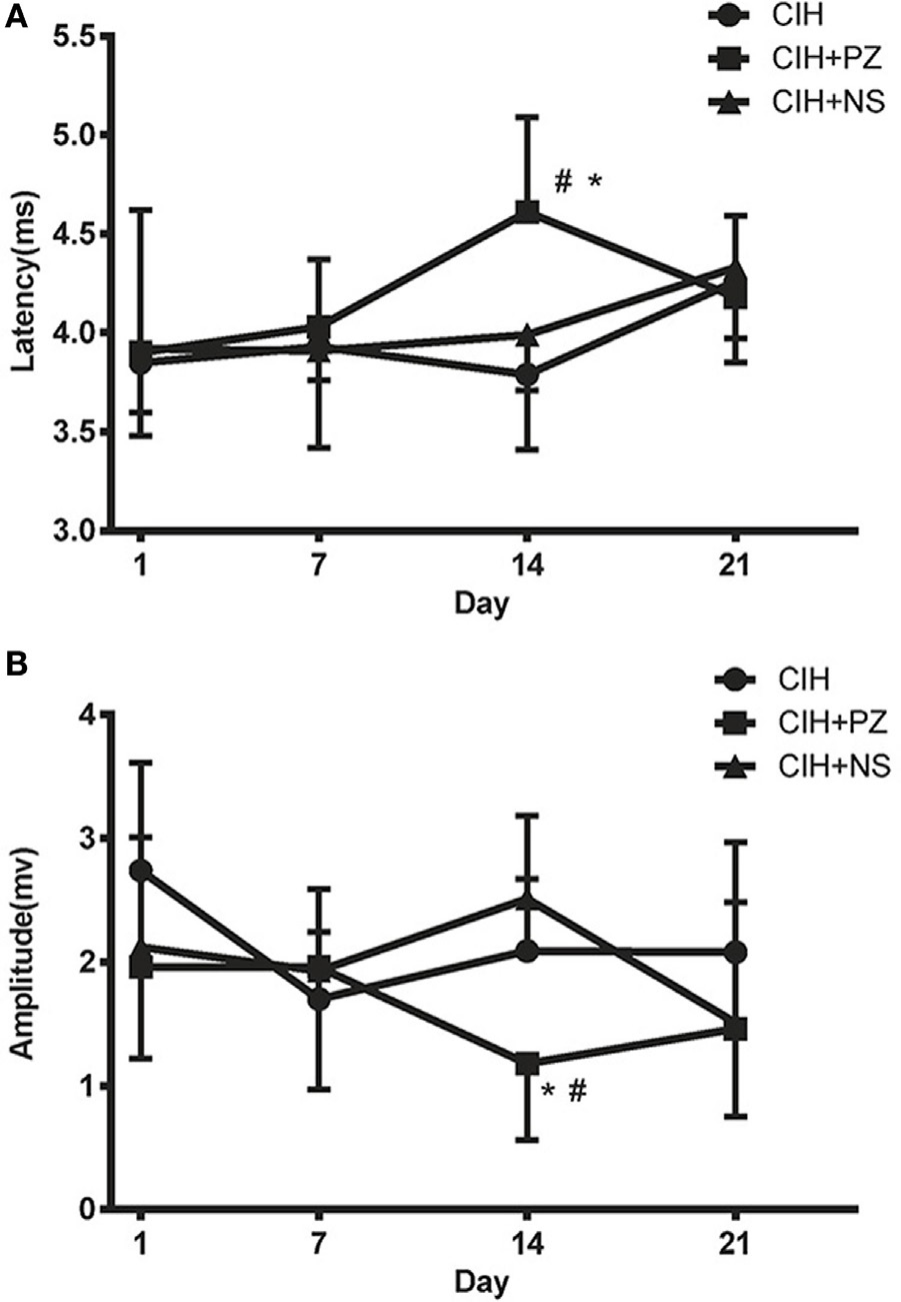
**The transcranial magnetic stimulation latencies (A) and amplitudes (B) of genioglossus corticomotor area in chronic intermittent hypoxia (CIH), CHI with prazosin (PZ) injection (CIH + PZ), and CHI with normal saline (NS) injection (CIH + NS) groups**. * indicates significant differences between CIH + PZ and CIH groups (*P* < 0.05). ^#^ indicates significant differences between CIH + PZ and CIH + NS groups (*P* < 0.05).

There was no difference in TMS responses between the CIH and CIH + NS groups. However, significantly longer MEP latencies and lower MEP amplitudes were observed in the CIH + PZ group than the CIH + NS group only on the 14th day of CIH (4.61 ± 0.48 vs. 3.99 ± 0.28 ms, 1.18 ± 0.62 vs. 2.51 ± 0.67 mV, *P* < 0.05) (Figures 3A,B). It showed that PZ microinjection inhibited CIH-induced excitability of genioglossus corticomotor area on the 14th day of exposure.

## Discussion

Many preliminary studies that investigated the role of noradrenergic activation of the hypoglossal nucleus indicated that α1-adrenoceptor mainly mediated the endogenous noradrenergic excitatory drive to the nucleus. However, only a few studies reported the effect of CIH on noradrenergic activation of hypoglossal motor neurons. Our studies in rats suggest noradrenergic activation of hypoglossal nucleus, through α1-adrenoreceptor participates in the central compensation of genioglossus during CIH. Furthermore, this is the first study to continuously observe the effects of noradrenergic activation of hypoglossal motor neurons on genioglossus corticomotor area at different stages of CIH.

Transcranial magnetic stimulation is a non-invasive functional approach that enabled us to evaluate the corticospinal pathway of genioglossus. In this study, it was used to assess the corticomotor activity of genioglossus during CIH. Compared to the NO group, the CIH group showed increased activity of the genioglossus corticomotor area from the 1st to the 14th day. These data indicated that the compensation of genioglossus corticomotor area occurred immediately after CIH and lasted for at least 2 weeks, which was consistent with the report that CIH could initiate respiratory plasticity (6). It is important to understand the mechanism of this compensation because it will be helpful to OSA treatment. Mateika et al. have reported that intermittent hypoxia may have therapeutic value in humans (15). Singh and Mallick provided evidence for a role of NE in central regulation of the genioglossus in REM-state (16). Withdrawal of NE in the hypoglossal nucleus of rats during REM state inhibited hypoglossal nerve activity (9). Chan et al. showed that the α1-adrenoceptor agonist, phenylephrine could increase genioglossus activity across sleep–wake states (17), whereas PZ injection into the hypoglossal nucleus decreased hypoglossal nerve output after three hypoxic episodes in rats (11). These findings raised the possibility that NE played its role in genioglossus control *via* α1-adrenoceptor in the hypoglossal nucleus during CIH. Stettner et al. found that on the 35th day of CIH, hypoglossal nerve activity significantly decreased in CIH-treated compared to sham-treated rats after PZ injection (12). However, there are no data on when NE regulates CIH-induced central compensation of the genioglossus, and how long its effect lasts. Our data on different stages of CIH demonstrated a significant effect of NE on the 14th day of CIH.

In the first week of CIH, PZ did not induce a change in responses of the genioglossus to TMS. However, a long MEP latency and low MEP amplitude could be observed in the second week of CIH. There is currently no explanation for this difference. Cao et al. have found that carotid body noradrenergic receptors were downregulated during 24–36 h of hypoxic exposure in cats (18). Wan and his colleagues showed that prolonged CIH could gradually increase plasma NE concentration, which positively correlated with the degree of hypoxia (19). Therefore, the absence of change in TMS in the CIH + PZ group might be caused by temporary downregulation of adrenergic receptors on hypoglossal nucleus at the beginning of CIH. Expressions of α1-adrenoceptor and NE terminal varicosities in hypoglossal nucleus were found to increase after 35 days of hypoxia (20). Su et al. indicated that TMS responses weakened from the 1st to the 28th day of CIH when a 5-HT neuronal damaging agent (anti-sert-SAP) was microinjected into raphe nucleus of rats (8). A previous study reported that both 5-HT and NE were necessary for expression of hypoglossal long-term facilitation induced by acute intermittent hypoxia (10). Inhibition of hypoglossal nucleus activity could be caused by combined withdrawal of NE and 5-HT on hypoglossal nucleus in REM sleep (9). Hence, we could hypothesize that during CIH, 5-HT, instead of NE played a major role in the early stages of CIH. With gradual passage of hypoxia, the endogenous NE drive might have recovered from being downregulated, to participate in the central compensation of genioglossus. It is noteworthy that, after injection of NE and 5-HT receptor antagonist into hypoglossal nucleus in normoxia, PZ induced more significant reduction of hypoglossal nerve activity than serotonin receptor antagonist, methysergide (19.1 ± 3.6 vs. 65.1 ± 6.6%) (9). Moreover, endogenous wakefulness-related drive to upper airway muscles mediated by NE was even more powerful than by 5-HT in rats (17). Noradrenergic disfacilitation was responsible for approximately 90% of the depression of hypoglossal motor neurons, whereas the remaining 10% could be explained by 5-HT in wakeful rats (21). Further research is needed to investigate whether endogenous NE drive is stronger than 5-HT on hypoglossal nucleus during CIH.

Ryan and Nolan suggested that the number of episodes of hypoxic exposure was a key determinant of long-term facilitation induced by hypoxia (22). The result of Tadjalli et al. indicated that more severe hypoxic level induced greater long-term facilitation of the genioglossus (23). Wang et al. showed that the enhanced central motor conduction time of the genioglossus correlated with apnea–hypopnea index, nadir oxygen saturation, and the longest apnea time of OSA patients (13). However, our experiment suggested that the increased TMS response disappeared on the 21st day of CIH. In the study by Stettner et al., microinjection of PZ into the hypoglossal nucleus induced increased endogenous NE drive after 35 days of CIH. Compared to our study, their experiment had a lower nadir oxygen concentration, more hypoxia cycles, and longer daily exposure time (lasted for 33–37 days, O_2_ level oscillated between 24 and 7%, 180-s period for 10 h/day) (12). In addition, NE was not the only neurotransmitter responsible for the facilitation of genioglossus central control. The role of 5-HT and other neurotransmitters needed to be explored. These may explain the discrepancy between the two experiments.

We should pay attention to the negative results following PZ microinjection into the hypoglossal nucleus during normoxia. In previous studies, three successive PZ microinjections were usually given at rostrocaudual level of the hypoglossal nucleus. This could elicit long-lasting changes in hypoglossal outputs for sustained observation (over 60 min) of the onset and peak time of endogenous NE drives (9, 10, 12). One focus of our study was to compare the onset time of TMS response in hypoxic and normoxic conditions and to confirm the increased reactivity and sensitivity of endogenous NE drive induced by CIH. Therefore, a single microinjection of PZ was given, and the measurement was completed within the 10-min half-life time of PZ (24). In our experiment, TMS response of genioglossus was not affected by PZ injection into the hypoglossal nucleus during air exposure, but there was significant difference between the CIH + PZ and CIH group. These data showed that excitatory drive of endogenous NE to the hypoglossal nucleus was triggered in CIH but not in normoxic rats. It was consistent with previous studies that CIH induced faster NE drive within 10 min (12).

**Figure 4:**
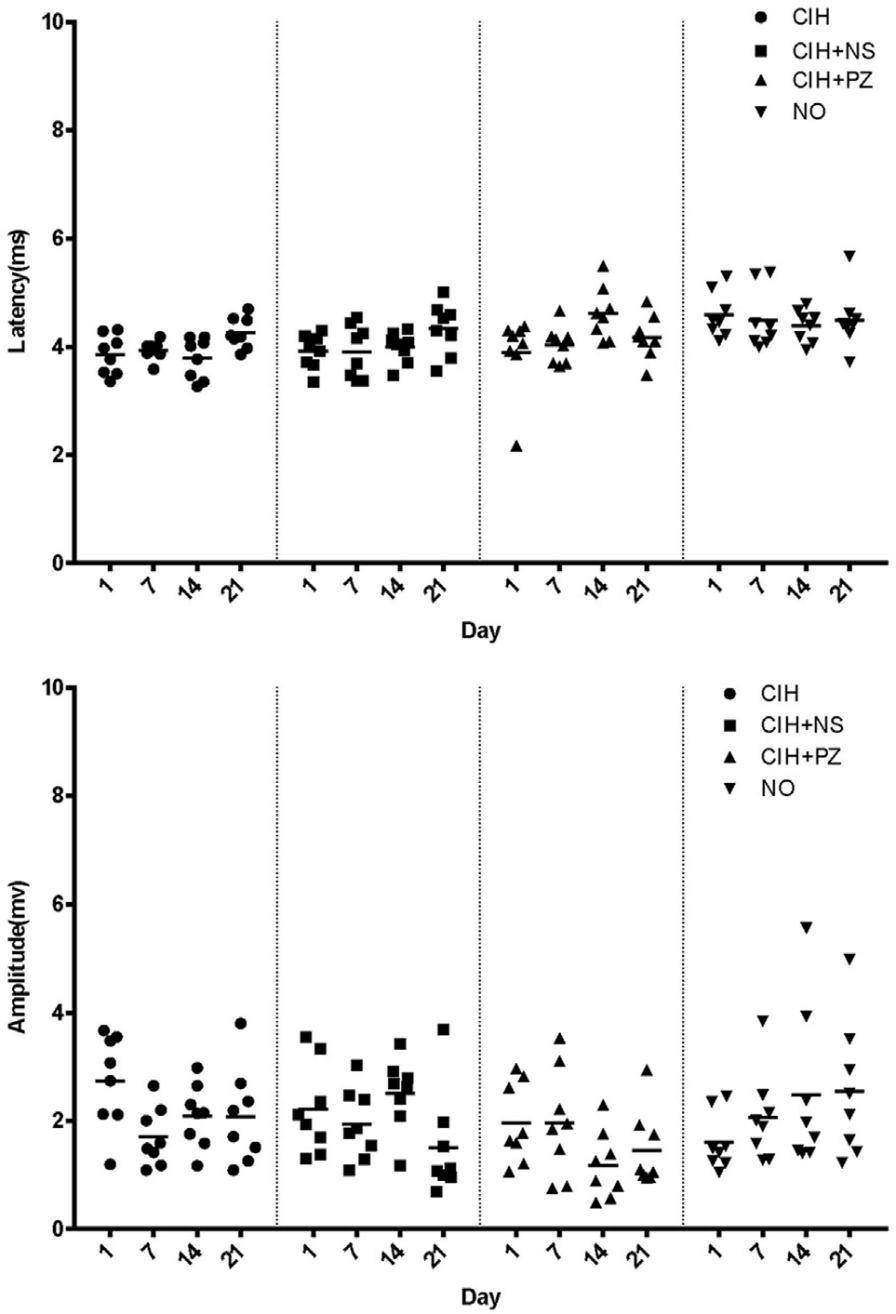
**Motor-evoked potential latencies and amplitudes of rats in chronic intermittent hypoxia (CIH) (*n* = 8), CIH + normal saline (NS) (*n* = 8), CIH + prazosin (PZ) (*n* = 8), and NO (*n* = 8) groups**.

There are still some limitations in our study. First, we only used male rats for this study. It had been reported that estrogen could partly reverse the contractile properties and fiber type distribution caused by CIH in rats (25). On the contrary, CIH could decrease the expression of estrogen receptor at both the mRNA and protein levels in rats (26). Thus, we only used male rats to exclude the influence of sex hormones on our outcome measures. Second, previous studies indicated that both 5-HT and NE participated in the atonia of hypoglossal motor neurons during CIH. We had explored the effects of 5-HT in our previous work. Therefore, we only focused on the influence of noradrenergic activation of hypoglossal motor neurons on the central control of genioglossus during CIH in the present study. The combined effect of 5-HT and NE, which is our next study question, remains unknown. Third, it was unfortunate that we could not demonstrate TMS response on a daily basis because we had to prevent the genioglossus from severe damage. However, we did observe a significant difference between CIH + PZ and CIH + NS groups on the 14th day. We also checked the raw data of each group, and there was no outlier to confound interpretation of the results (Figure 4).

Singh and Mallick observed the exact effect of NE in physiological processes caused by loss of REM-stage and suggested the levels of NE in the brain as a target for treatment of REM stage-related diseases (16). OSA is observed only during sleep in the presence of neuromuscular compensation. On the basis of this compensation, some investigators have tried to establish new methods to alleviate OSA. This study revealed that noradrenergic activation of hypoglossal nucleus played an important role in the central compensation of genioglossus through α1-adrenoceptor on the 14th day of CIH. It suggested that NE contributed to the increased upper airway stability at the moment of CIH. Understanding the mechanisms that regulate upper airway stability during CIH is key for finding new pharmacological targets to treat OSA.

## Ethics Statement

Procedures and experiment protocols were performed in accordance with the National Institute of Health Guide for Care and Use of Laboratory Animals and were approved by the animal Ethics and Use Committee of China Medical University.

## Author Contributions

XN: TMS performance, data analysis, manuscript drafting, and final approval of the version to be published. LZ: whole experiment performance, data analysis, manuscript drafting, and final approval of the version to be published. AW and JP: in charge of microinjection and TMS experiment, data analysis, manuscript drafting, and final approval of the version to be published. HJ: in charge of in intermittent hypoxia and microinjection experiment, data analysis, and final approval of the version to be published. ZQ: in charge of intermittent hypoxia experiment, data analysis, and final approval of the version to be published. WW and JK: study design, data interpretation, critical manuscript revision, and final approval of the version to be published.

## Conflict of Interest Statement

The authors declare that the research was conducted in the absence of any commercial or financial relationships that could be construed as a potential conflict of interest.

## Abbreviations

AHI, apnea–hypopnea index; CIH, chronic intermittent hypoxia; EMG, electromyogram; MEP, motor-evoked potential; NE, norepinephrine; NO, normoxic group; NS, normal saline; OSA, obstructive sleep apnea; PBS, phosphate-buffered saline; PZ, prazosin; REM, rapid eye movement; TMS, transcranial magnetic stimulation; 5-HT, 5-hydroxytryptamine.
